# A case report of malignant transformation in neurofibromatosis type 1: pain and rapid growth as key indicators for early biopsy

**DOI:** 10.3389/fonc.2026.1800068

**Published:** 2026-04-07

**Authors:** Yuan Zhao, Jun-ya Cao, Yuqian Li, Jun-Yi Huo, Zhuo-Wei Zhao, Le Xue, Jing Li, Ce-Zhong Duan

**Affiliations:** Department of Burns and Plastic Surgery, Tangdu Hospital, The Fourth Military Medical University, Xi’an, Shaanxi, China

**Keywords:** case report, literature review, malignant peripheral nerve sheath tumors, neurofibromatosis type I, plexiform neurofibroma

## Abstract

Malignant peripheral nerve sheath tumor (MPNST) represents a life-threatening complication of neurofibromatosis type 1 (NF1). This report describes a male patient with NF1 and concomitant MPNST treated at our institution. A 55-year-old male first presented with multiple, skin-colored neoplasms distributed across his body over the course of three decades, without any identifiable predisposing factors. Two years before presentation, a firm subcutaneous nodule appeared on his left back, causing intermittent mild discomfort, leading to a diagnosis of multiple neurofibromas. Despite participation in a clinical trial, the lesion on his left back progressively enlarged, and associated pain intensified. A multidisciplinary team subsequently recommended resection of the dorsal mass via Mohs surgery under general anesthesia. Postoperative histopathological analysis confirmed NF1 with concurrent MPNST. No adjuvant targeted therapy was administered, and no recurrence was observed during the six-month follow-up. This case highlights the coexistence of NF1 and MPNST, aiming to enhance clinical awareness of the malignant transformation risks in NF1, thereby promoting earlier diagnosis and minimizing the potential for misdiagnosis and delayed intervention.

## Introduction

Neurofibromatosis type 1 (NF1) is an autosomal dominant genetic disorder resulting from mutations in the NF1 gene (1), with a minimum estimated prevalence of 1 in 3,000 to 1 in 4,000 individuals and an incidence of 1 in 2,500 live births (2). Clinically, NF1 is characterized by multiple flat, light-brown cutaneous pigmentary patches, skinfold freckling, subcutaneous neurofibromas, and iris hamartomas (3). Currently, no curative treatment exists for NF1, and management is limited to symptomatic interventions aimed at alleviating local manifestations, including laser therapy, surgical procedures, targeted drug therapy, and treatment of complications (4). Individuals with NF1 have a reduced life expectancy, averaging 15 years less than the general population (3). Therefore, improving clinical awareness of NF1’s natural history, optimizing surveillance for potential complications, and advancing targeted therapeutic strategies are essential to improve patient outcomes and mitigate the long-term impact of the disease on survival and quality of life (QoL).

Malignant peripheral nerve sheath tumor (MPNST) is a relatively rare neoplasm, accounting for 5–10% of all soft-tissue sarcomas, characterized by nerve sheath differentiation and high metastatic potential (5). Its lifetime incidence in the general population is approximately 0.001% (1 in 100,000 individuals) (6). Despite its low incidence, MPNST is associated with poor prognosis, with a 5-year survival rate of only 30% (5). Notably, 50% of MPNST cases occur in individuals with NF1, with a mean age of onset between 20 and 40 years (7). MPNST is the leading cause of mortality in NF1 patients (8), with unfavorable outcomes attributed to delayed diagnosis and, more significantly, the limited success of therapeutic interventions. Although complete surgical resection is the primary treatment for MPNST, it is often complicated by large tumor size, proximity to complex nerve structures, and low rates of negative resection margins (9, 10). However, biological complexities such as recurrence, metastasis, and mortality in MPNST remain incompletely understood due to small patient cohorts, limited large-scale clinical studies, and variability in survival data. Moreover, the majority of published studies focus on the clinicopathological characteristics of patients (11). This case report of a 55-year-old male with NF1 and concurrent MPNST highlights the importance of recognizing pain and rapid tumor growth as potential indicators of malignant transformation in NF1, warranting timely biopsy and multidisciplinary management.

## Case description

A 55-year-old male patient first developed multiple scattered nodules, approximately the size of mung beans, across his body over 30 years ago, without any identifiable predisposing factors. These nodules, which ranged in color from skin-toned to pale purple-red, were soft in texture and asymptomatic (without pain or pruritus), and no medical attention or treatment was sought at that time. Over the years, the skin lesions progressively increased in both size and number. Two years prior to presentation, a firm subcutaneous nodule appeared on his left back, accompanied by occasional pain. The patient sought evaluation at another hospital, where the lesion was diagnosed as “multiple fibromas,” though no specific intervention was provided. Subsequently, the nodule on his left back continued to enlarge, and the pain intensified. He returned to the external hospital, where MRI findings were consistent with neurofibromatosis. Genetic testing confirmed an NF1 gene mutation (c.4923G>A). After evaluation and approval, the patient enrolled in a randomized, double-blind, placebo-controlled, multicenter Phase III clinical trial assessing the efficacy and safety of FCN-159 in adults with symptomatic, inoperable plexiform neurofibromas (pNFs) associated with NF1. During treatment, he developed facial swelling and ulceration; concurrently, the lesion on his left back further enlarged, and the pain worsened, severely impacting his QoL, prompting him to seek care at our hospital. The patient had no history of major internal diseases or infectious conditions and denied a family history of hereditary disorders.

## Diagnostic assessment

**Figure 1:**
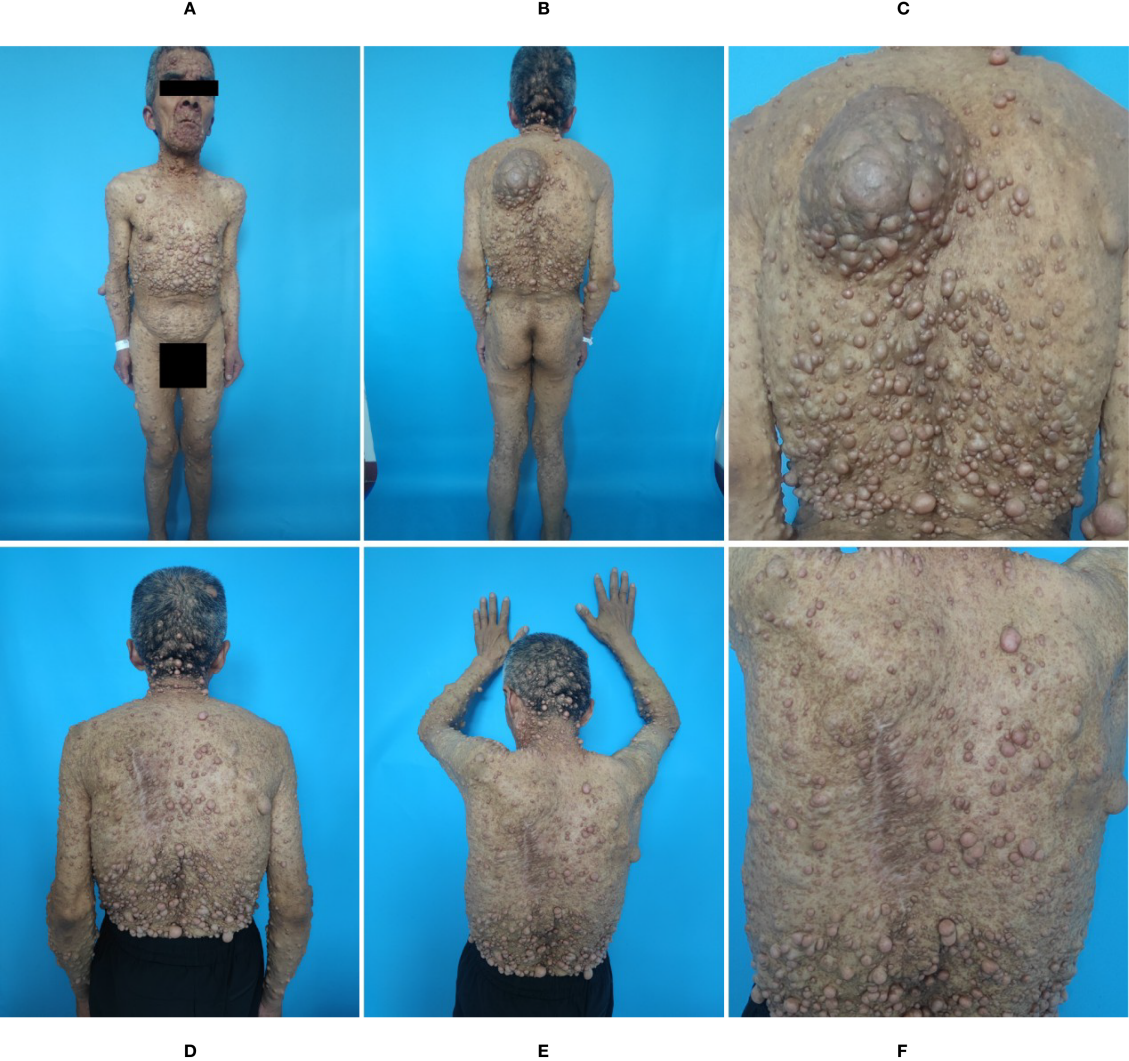
**(A-C)** At admission, ‘Café-au-lait’ spots and nodules over the patient’s entire body, with a hard, slightly erythematous, irregularly shaped, elevated tumor with local surface ulceration. **(D-F)** At 3 months after surgery, no recurrence of the tumors was observed on the patient’s chest and back, and the motor function of both upper limbs remained well-preserved.

Dermatological examination revealed scattered tan patches across the body of varying sizes, with irregular shapes, well-defined borders, and uniform coloration. Numerous skin-colored nodules, of varying sizes, were densely distributed over the entire body, with well-defined borders and soft texture. Notably, a larger lesion on the left back, measuring approximately 15 cm × 10 cm, was firm to the touch and tender on palpation (**Figure 1**).

**Figure 2:**
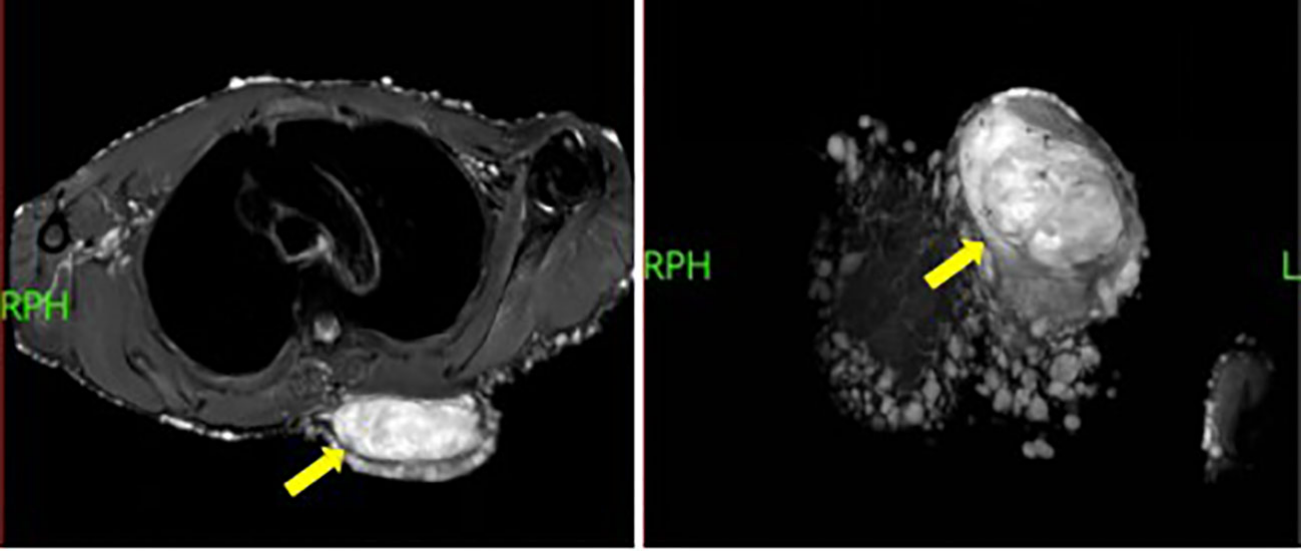
MRI contrast-enhanced scan showing nodular and mass-like shadows. The arrow indicates a mass on the left side of the chest and back.

Auxiliary investigations: Chest CT showed uneven thickening of the skin over the bilateral thoracic cage, shoulders, and neck, with multiple nodules and mass-like opacities, consistent with neurofibromatosis (**Figure 2**). Needle biopsy revealed a spindle cell lesion, suggestive of a neurogenic tumor. Combined with histopathological features and clinical characteristics, the findings were consistent with neurofibromatosis.

Based on the revised diagnostic criteria for NF1 proposed by the International Neurofibromatosis Diagnostic Criteria Consensus Group in 2021, the patient met the criteria for NF1. After a multidisciplinary consultation (including input from the oncology department) and exclusion of contraindications, the patient underwent Mohs surgery under general anesthesia to resect the dorsal mass. Intraoperative pathology confirmed negative margins, and the surgical site was closed using an ultra-tension-reducing suture technique. Postoperatively, targeted anti-tumor therapy with selumetinib was recommended, and continued participation in clinical trials was encouraged.

**Figure 3:**
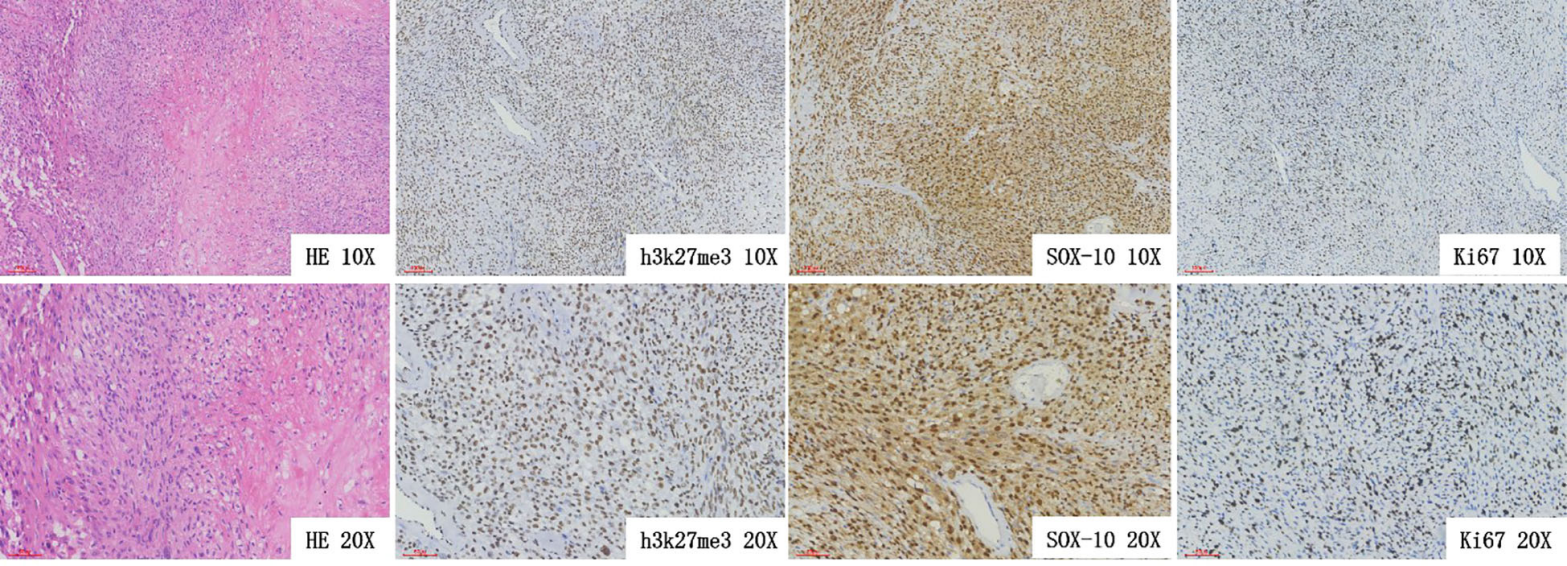
Histopathological examination revealed a spindle cell lesion with marked local cell proliferation activity. Combined with the histological morphology and immunophenotypic profiles, the lesion was diagnosed as consistent with malignant peripheral nerve sheath tumor (MPNST).

Postoperative pathology (Left back mass): Neurofibroma with focal areas of highly active cellular proliferation; combined with histopathological features and immunophenotypic characteristics, the findings were consistent with MPNST (**Figure 3**). At the 6-month follow-up, no tumor recurrence was observed at the surgical site, and no sensory or motor deficits were noted in the limbs.

## Discussion

NF1 is a rare neurogenetic disorder marked by multifocal organ involvement and a heightened susceptibility to both benign and malignant neoplasms (12). Diagnosis is based on clinical criteria, with genetic confirmation supporting the diagnosis, following updated guidelines from the International Neurofibromatosis Diagnostic Criteria Consensus Group in 2021 (13). In individuals without a family history of NF1, diagnosis requires the presence of at least two of the following seven clinical features: (1) ≥ 6 café-au-lait spots, with a maximum diameter > 5 mm before puberty and > 15 mm after puberty; (2) axillary or inguinal freckling; (3) ≥ 2 neurofibromas of any type or one pNF; (4) optic pathway glioma; (5) ≥ 2 iris Lisch nodules or choroidal abnormalities detected by slit-lamp examination; (6) characteristic bony lesions such as sphenoid dysplasia, anterolateral tibial bowing, or pseudarthrosis of long bones; (7) a heterozygous pathogenic NF1 variant with a 50% variant allele fraction in normal tissue. In this case, the patient had no family history but met the diagnostic criteria for items (1) and (3), and gene sequencing confirmed a heterozygous mutation in the NF1 gene, confirming the diagnosis of NF1. NF1 affects multiple organs and tissues, presenting with both neoplastic and non-neoplastic manifestations (14). The clinical diagnosis of NF1 is made when two or more of the following features are present: six or more café-au-lait macules, skinfold freckling, Lisch nodules, characteristic bony lesions, optic pathway gliomas, cutaneous or deep neurofibromas, or a first-degree relative with NF1 (15). Neurofibromas are among the most common manifestations in affected individuals. These tumors primarily consist of neoplastic Schwann cells but also contain a variety of soft tissue and nerve components, including perineurial cells, axons, mast cells, and fibroblasts, which contribute to tumor growth. Cutaneous neurofibromas are the predominant form and may be numerous. Of greater concern are large pNFs, which have a propensity to undergo malignant transformation into MPNST, a leading cause of mortality in NF1 patients. Ocular manifestations also play a pivotal role in the diagnosis of NF1. Lisch nodules, which are asymptomatic hamartomatous aggregates of melanin-containing cells on the iris, are present in nearly all patients and can be assessed via comprehensive ophthalmologic examination. These varied manifestations highlight the complexity of NF1 as a multisystem disorder, emphasizing the need for multidisciplinary surveillance to monitor disease progression and address malignant transformation risks (14).

At the molecular level, the clinical complexity of NF1 is rooted in the functions of the NF1 gene and its encoded protein. The NF1 gene, located on chromosome 17q11.2 and spanning approximately 60 exons, encodes neurofibromin, a GTPase-activating protein expressed in various cell types, including neurons, astrocytes, and oligodendrocytes (14). Individuals with NF1 inherit one inactivated NF1 allele, and tumor development occurs upon loss of the second allele (16). Genetic screening for NF1 faces significant challenges due to the gene’s large size, extensive diversity of pathogenic variants, and limited genotype-phenotype correlations (17). Tsipi et al. recently identified 70 novel alterations using peripheral blood-based next-generation sequencing (NGS) and multiplex ligation-dependent probe amplification (MLPA) (17). Although large NF1 deletions may correlate with more severe phenotypes and central nervous system (CNS) structural anomalies (18), this association remains under investigation. Neurofibromin’s primary function is the direct inhibition of RAS signaling by converting active GTP-bound RAS to its inactive GDP-bound state (14). Inactivation of NF1 results in uncontrolled RAS signaling, driving mitogen-activated protein kinase (MAPK) pathway activation via RAF, extracellular signal-regulated kinases 1/2 (ERK1/2), and subsequent transcriptional activation and cell growth (14). Additionally, unregulated RAS cross-activates the PI3K-mTOR pathway: GTP-bound RAS activates PI3K, promoting cell survival and proliferation via AKT/mTOR, while ERK-mediated phosphorylation of TSC2 further enhances mTOR activity (19). This multi-pathway mechanism contributes to tumorigenesis and neurodevelopmental abnormalities due to neurofibromin loss. Beyond RAS regulation, neurofibromin also influences cAMP signaling. Nf1-deficient models (drosophila, zebrafish, mice) demonstrate reduced brain cAMP levels (20). These insights into cAMP-dependent mechanisms broaden the therapeutic landscape for NF1, highlighting the need for pathway-specific investigations to address both tumorigenic and neurodevelopmental aspects of the disorder.

MPNSTs are rare sarcomas, accounting for 5–10% of all soft tissue sarcomas (5). These aggressive malignancies of the nervous system arise either *de novo* from normal neural components, such as Schwann cells and perineural cells, or from pre-existing benign peripheral nerve sheath tumors. MPNSTs most commonly develop in the extremities, presenting as painful masses that may be accompanied by neurologic symptoms (21). Most MPNSTs are high-grade sarcomas with a strong propensity for recurrence (40–65%) and metastasis (40–80%) (22). Prognosis remains poor, with 5-year survival rates as low as 30–60% in modern series for patients with localized MPNST following attempted curative resection (23). Currently, there is no highly accurate diagnostic standard for MPNST (24), and due to the strong similarities in symptoms and imaging with other soft tissue tumors, distinguishing MPNSTs can be challenging (25). Multiple genetic alterations contribute to MPNST pathogenesis, including aberrations in cyclin-dependent kinase inhibitor 2A (CDKN2A), phosphatase and tensin homolog (PTEN), SUZ12, epidermal growth factor receptor (EGFR), platelet-derived growth factor receptor (PDGFR), and MET (21). Complete R0 surgical excision remains the only curative treatment for localized MPNST. However, even after complete resection, these tumors exhibit high rates of local recurrence and/or metastasis (21). Radiation therapy and systemic therapies may be considered as risk-reduction modalities in neoadjuvant or adjuvant settings, although their use remains controversial due to the lack of definitive data demonstrating significant risk modification in MPNST patients (21). Despite this uncertainty, multimodal treatment approaches have shown improved survival outcomes compared to historical controls, emphasizing the importance of referral to specialized sarcoma centers for multidisciplinary treatment planning (26).

The leading cause of early mortality in patients with NF1 is MPNST. MPNSTs most commonly arise from pNFs or nodular neurofibromas, and individuals with NF1 have a lifetime risk of 8–13% for developing an MPNST (27). In contrast, sporadic MPNSTs are more frequently observed in older patients compared to those with NF1, with many patients with NF1 developing MPNSTs during adolescence or young adulthood (28). While controversial, some data suggest that MPNSTs occurring in the context of NF1 syndrome are associated with poorer clinical outcomes (29). A retrospective study of 395 NF1 patients conducted in the early 2000s by Karen Leroy demonstrated that MPNSTs exhibit high aggressiveness in NF1, typically arising from plexiform or nodular neurofibromas. The study emphasized the importance of investigation and deep biopsy for painful or enlarging neurofibromas in NF1 patients (30). Moreover, neoadjuvant chemotherapy has been shown to prevent limb amputation, facilitate extensive tumor resection, restore limb function, and significantly improve physical condition (31). In another case, a 71-year-old female with a family history of NF1-associated epithelioid MPNST derived from peripheral nerves underwent surgery and subsequent adjuvant radiotherapy (60 grays). No abnormalities were observed in post-treatment imaging, and there was no recurrence or metastasis within four years (32). HPNSTs are frequently observed in association with tumor syndromes, notably NF2-related schwannomatosis, other schwannoma variants, and less commonly, NF1 (33, 34).This case indicates favorable outcomes in selected NF1-associated MPNSTs treated with combined surgery and adjuvant radiotherapy, supporting the value of multidisciplinary care and prolonged surveillance.

Preoperative assessment is critical for determining postoperative functional changes. Tools such as the Highet Muscle Strength Grading, Visual Analog Scale (VAS), and Douleur Neuropathique 4 Questions (DN4) Scale are effective for preoperative evaluation in NF1 patients (35). For instance, a 2020 study on NF1-associated scoliosis found that patients with preoperative neurological deficits and more dystrophic features were at a higher risk of intraoperative neurophysiological monitoring (IONM) failure, providing a basis for individualized preoperative surgical planning (36). Conversely, re-evaluating muscle strength, pain, and sphincter function within 48 hours postoperatively can help identify new-onset neurological deficits. Mid- to long-term postoperative recovery can be assessed through a combination of tools, such as nerve conduction velocity (NCV) and electromyography (EMG), the Semmes-Weinstein monofilament test, the two-point discrimination test, the House-Brackmann Scale for facial nerve palsy recovery, neurological function-related QoL scales, and biochemical marker measurements (37, 38). While no postoperative complications—such as tumor recurrence or limb sensory and motor dysfunction—were observed in the patients of this study, long-term follow-up remains essential to monitor for delayed neurological changes or disease progression.

Despite the positive outcomes in the highlighted cases, significant challenges remain in clinical practice. Early detection of NF1-associated MPNST is particularly difficult due to the absence of specific symptoms, and distinguishing malignant transformation (e.g., pain, rapid enlargement) from benign growth can be clinically ambiguous, leading to delays in biopsy and intervention (39). Given that NF1-associated MPNSTs are Ras-driven due to neurofibromin loss, targeted inhibition of downstream pathways such as MAPK and PI3K represents a critical area of research (40). Preclinical studies have demonstrated that MEK inhibitors are effective in reducing tumor growth; however, clinical translation is hindered by resistance mechanisms and toxicity (41). Moreover, the unique histological and molecular features of epithelioid MPNSTs necessitate subtype-specific investigations to uncover therapeutic vulnerabilities, such as potential targets specific to the epithelioid cell phenotype (32). The present study is a single-case report, and the postoperative follow-up period was relatively short. Consequently, long-term disease-free survival and functional outcomes require further follow-up and observation. Addressing these gaps through biomarker development, refined targeted therapies, and subtype-tailored investigations will be crucial for advancing precision care and improving outcomes in this high-risk patient population.

In conclusion, NF1 with concurrent MPNST is a rare and complex disease that demands early identification, accurate diagnosis, multidisciplinary collaboration, individualized treatment, and rigorous surveillance to improve patient outcomes. Despite incremental progress in combining targeted and chemotherapeutic approaches, overall prognostic outcomes remain comparable to those of conventional therapies. Advances in genomic research have expanded diagnostic and therapeutic options for neurofibromatosis. The identification of novel therapeutic targets, development of effective treatment protocols, and innovation of validated strategies could potentially transform future management approaches, improving survival rates and QoL for patients with NF1. Through the presentation of this case and a review of the literature, our understanding of NF1 can be enhanced, and heightened vigilance regarding malignant progression can facilitate earlier recognition, accurate diagnosis, and prevent misdiagnosis or delayed intervention.

## Patient perspective

I am grateful for the timely diagnosis and skilled treatment I received. This experience has taught me to be vigilant about changes in my skin lesions. For NF1 patients, pain and rapid tumor growth should never be ignored. I hope my story raises awareness, encouraging others to seek prompt medical attention to avoid delayed diagnosis and treatment.

## Data Availability

The original contributions presented in the study are included in the article/**Supplementary Material**. Further inquiries can be directed to the corresponding author.

## Supplementary material

Supplementary Figure 1 Timeline with relevant data from the episode of care.
