# Supplementary figures and images for: A case report of malignant transformation in neurofibromatosis type 1: pain and rapid growth as key indicators for early biopsy
Source: Front Oncol. 2026 Apr 7;16:1800068. doi: 10.3389/fonc.2026.1800068 (PMC13095573; doi:10.3389/fonc.2026.1800068)

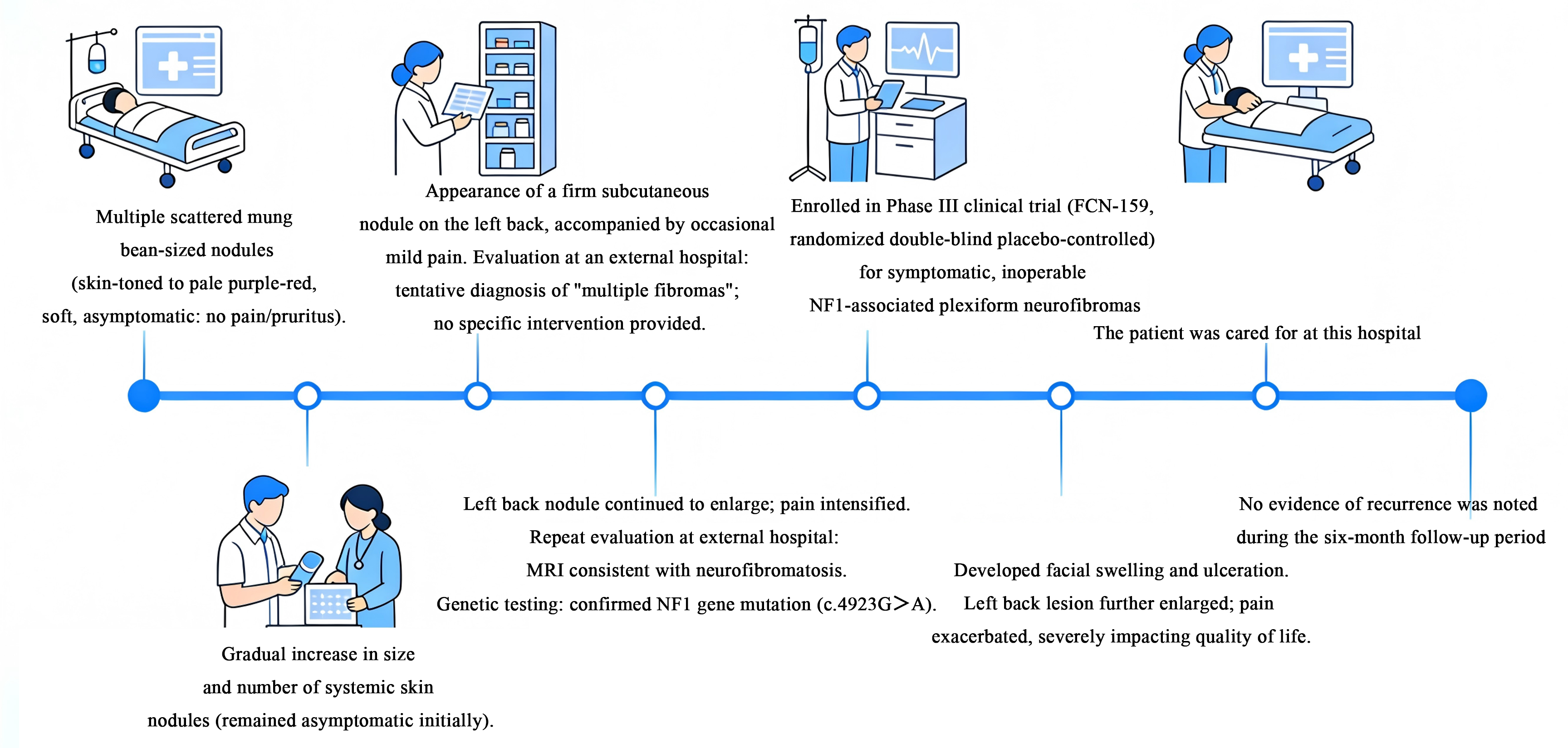

Supplement: Supplementary Figure 1 — Timeline with relevant data from the episode of care. [file Image1.tif]
